# Impact of craniocervical junction abnormality on vertebral artery hemodynamics: based on computational fluid dynamics analysis

**DOI:** 10.3389/fneur.2023.1244327

**Published:** 2024-01-05

**Authors:** Zeyuan Zhang, Xin Ai, Yuanzhi Xu, Yuqiang Wang, Shuhao Zhang, Yao Zhao, Ruifang Zhou, Rui Tang, Limin Wang, Yilin Liu

**Affiliations:** ^1^Department of Orthopedics, The First Affiliated Hospital of Zhengzhou University, Zhengzhou, China; ^2^School of Mathematics and Information Sciences, Zhongyuan University of Technology, Zhengzhou, China

**Keywords:** craniocervical junction abnormality, vertebral artery, computational fluid dynamics, computed tomography angiography, dizziness

## Abstract

**Background and purpose:**

A three-dimensional reconstruction and data analysis of the vertebral artery (VA) with craniocervical junction abnormality (CJA) was performed by computational fluid dynamics (CFD) based on images to assess the impact of CJA on vertebral artery hemodynamics.

**Methods:**

Retrospective analysis of combined head and neck computed tomography angiography (CTA) images of 60 patients with CJA and 60 normal patients admitted to our department from January 2018 to June 2022. The VA was reconstructed in three dimensions using CFD-related software, and the results were visualized to derive vertebral artery lumen diameter (D), peak systolic velocity (PSV), mean blood flow velocity (MV), wall pressure (P), wall shear stress (WSS), normalized WSS (NWSS), etc. Statistical analysis was used to analyze whether the data related to hemodynamics in the CJA group and the control group were statistically significant.

**Results:**

The lumen diameter of the vertebral artery in the CJA group were less than the control group, and the difference was statistically significant (3.354 ± 0.562 vs. 3.744 ± 0.520, *p* < 0.05); the PSV, MV, P, WSS, and NWSS of the CJA group were increased compared with the control group, and the difference was statistically significant (1.235 ± 0.182 vs. 1.104 ± 0.145, 0.339 ± 0.063 vs. 0.307 ± 0.042, 24576.980 ± 7095.836 vs. 20824.281 ± 6718.438, 34.863 ± 6.816 vs. 31.080 ± 5.438, 0.272 ± 0.075 vs. 0.237 ± 0.067, *p* < 0.05).

**Conclusion:**

In the complex CJA, the possibility of hemodynamic variation in the VAs is higher than in the normal population. The hemodynamic aspects of the vertebral artery in patients with CJA, such as diameter, flow velocity, flow, wall pressure and shear force, differ from those in the normal population and may lead to the occurrence of clinical symptoms, such as dizziness, so preoperative examinations such as combined head and neck CTA should be performed to clarify the vascular abnormalities.

## Introduction

Craniovertebral junction abnormalities (CJA) are abnormal pathological changes in the bones, soft tissues and nervous system of the occipital, atlanto-axial and cardinal spine (1). The deformity of the craniocervical junction (CVJ) area results in local anatomical diversity and complexity, and the area contains important structures such as the spinal cord and VA (2). CJA can lead to tortuous travel of the VA, causing changes in the hemodynamics of the blood vessels, aggregation and shedding of platelets, which in severe cases can lead to thrombosis and endanger the lives of patients. When there is a complex CJA, the corresponding cervical vascular variation is more likely to occur than in the normal population. At the same time, VA abnormalities may be correlated with posterior circulation ischemia, so patients with CJA may experience a series of VA-related symptoms, such as dizziness and headache, facial numbness, visual impairment and unsteadiness in walking, which are mainly caused by posterior circulation ischemia. Therefore, understanding the impact of CJA on VA hemodynamics is important to assess the patient’s condition and to surgical planning.

In patients with CJA, a combined head and neck CTA, MRA and DSA can be used to assess vascular status before surgery. Masashi Yamazaki (3) found that using preoperative 3D head and neck CTA can precisely identify anomalous VAs and thereby reduce the risk of intraoperative injury. However, existing imaging methods have limited resolution and are not intuitive enough for complex anatomical structures in the CVJ. Hence we explored different methods to clarify hemodynamic alterations based on imaging, named image-based computational fluid dynamics (CFD). CFD can simulate and analyze fluid mechanics problems that include fluid flow and heat conduction and other related physical phenomena through computer numerical calculation and image display based on imaging (4). The CFD technique allows the hemodynamic data to be obtained and statistically analyzed, while visualizing the changes in vertebral artery hemodynamics.

Several studies investigated the impact of cervical spondylosis on vertebral artery blood flow (5, 6), but few previous studies addressed the impact of CJA, specifically on vertebral artery hemodynamic parameters.

In this study, we performed 3D reconstruction and data analysis of VAs with CJA by CFD to assess the impact of CJA on VAs’ hemodynamics and the correlation between vertebral artery hemodynamic changes and patient symptoms, and to provide suggestions for clinical assessment and treatment planning for CJA patients.

## Materials and methods

### Patient data

Sixty patients with CJAs admitted to our department from June 2018 to February 2022 were selected for screening and used as the experimental group (CJA group), and 60 cases from the normal population were selected as the control group. The inclusion criteria were as follows: (i) those who meet the diagnostic criteria of CJA (CJA group); (ii) those who have complete imaging data of combined head and neck CTA; (iii) those who have informed consent from patients and family members for hemodynamic testing and approved by the hospital ethics committee. Combined severe underlying disease, combined primary related disease of the VA, and pregnant and lactating women were excluded (Table 1).

**Table 1:** Clinical data of the 120 study cases.

	CJA group	Control group	*t*	*P*
Total class	60	60		
Gender				
Male	26	35	−1.648	0.102
Female	34	25		
Age	48.70 ± 16.56	47.47 ± 11.26	0.469	0.64
Height	162.20 ± 6.34	164.57 ± 7.62	−1.848	0.067
Weight	59.02 ± 10.27	62.38 ± 10.99	−1.699	0.092
BMI	22.49 ± 3.34	22.37 ± 3.07	−0.850	0.397

The difference in basic information between the patient and control groups was not statistically significant (*P* > 0.05).

The CJA group included 33 cases of Atlantoaxial dislocation (AAD), 22 cases of Basilar invagination (BI), 14 cases of Os odontoideum, 19 cases of C1 occipitalization of the atlas, 10 cases of Sandwich deformity (occipitalization of the atlas with C2/3 fusion). The CJA group mostly had more than one of these deformities at the same time.

### 3D reconstruction and CFD simulation

**Figure 1:**
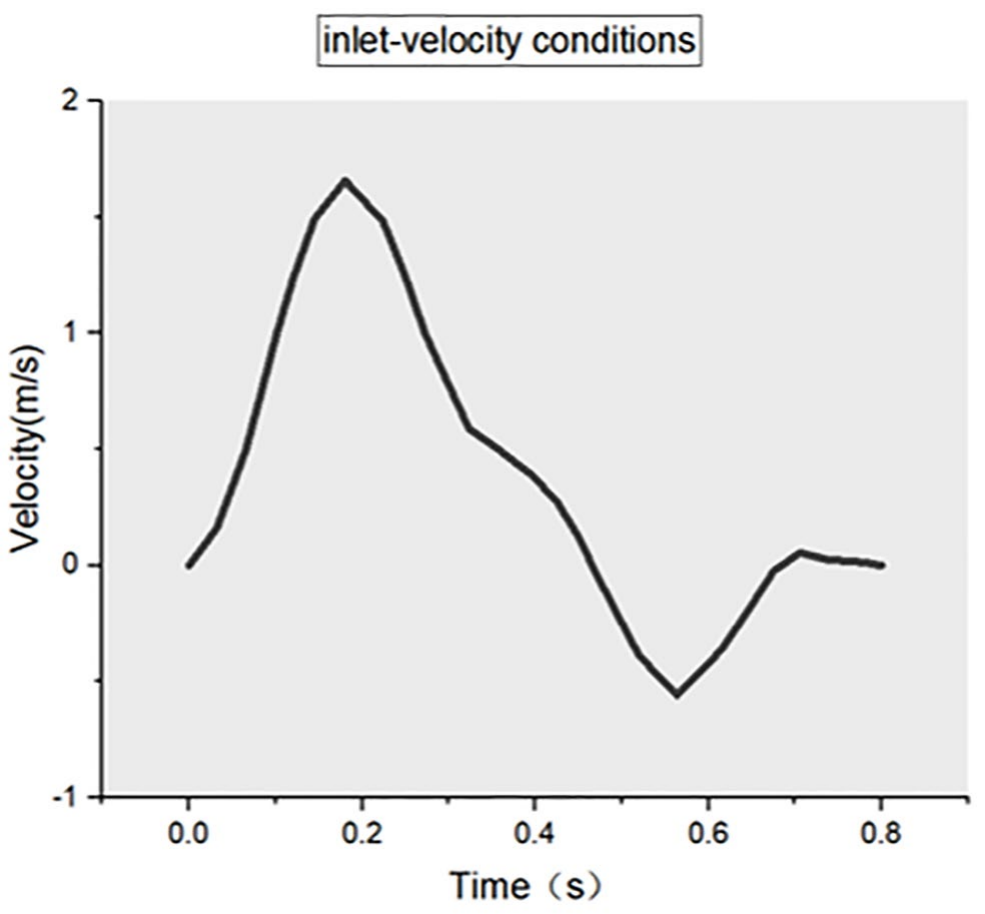
Inlet-velocity conditions.

**Figure 2:**
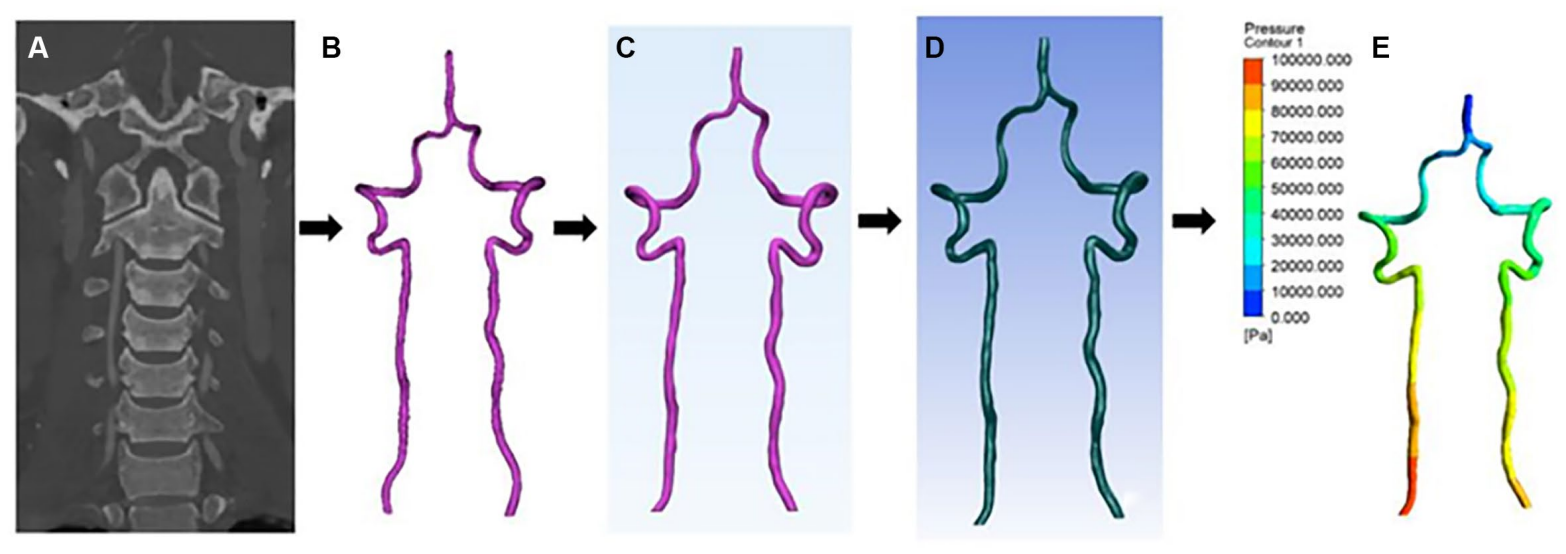
CFD reconstruction process. **(A)** Combined head and neck CTA coronal images. **(B–D)** Vertebral artery 3D model smoothing and meshing process. **(E)** CFD results visualization, the figure illustrates the change in pressure gradient at the wall of the vertebral artery.

Extracting of patient-specific vertebral artery geometry information from combined head and neck CTA and importing these CTA images (DICOM format) into the software Mimics Medical 21.0 (Materialise, Belgium). The 3D reconstruction of the patient-specific vertebral artery was obtained using automatic threshold segmentation and manual separation. Next, the arterial model was imported into 3-matic Medical 13.0 (Materialise, Belgium) for smoothing, cropping, and wall separation to obtain a CAD model of the VA. Then, the CAD model generated above was imported into Fluent Meshing (ANSYS, United States) for meshing, and the mesh density was refined at the bend of the VA to obtain the overall mesh model of the vertebral artery (MSH format). Finally, the resulting vertebral artery MSH mesh model was imported into Fluent 19.2 (ANSYS, United States) to set the initial conditions and boundary conditions. We assume that the blood flow is an incompressible, constant Newtonian fluid with a blood density of ρ = 1.04 × 10^3^ kg/m^3^ and a dynamic blood viscosity of 3.5 × 10^−3^ Pa·s. In the boundary condition settings, the two inlets are set to velocity inlet (Figure 1), the outlet is set to pressure outlet, and the numerical calculation is set to unsteady transient calculation, taking the normal human cardiac period of 0.8 s and time step of 0.01 s. The CFD results are visualized using CFD-Post 19.2 (ANSYS, United States) and the related data are exported (Figure 2).

### Hemodynamic data acquisition

**Figure 3:**
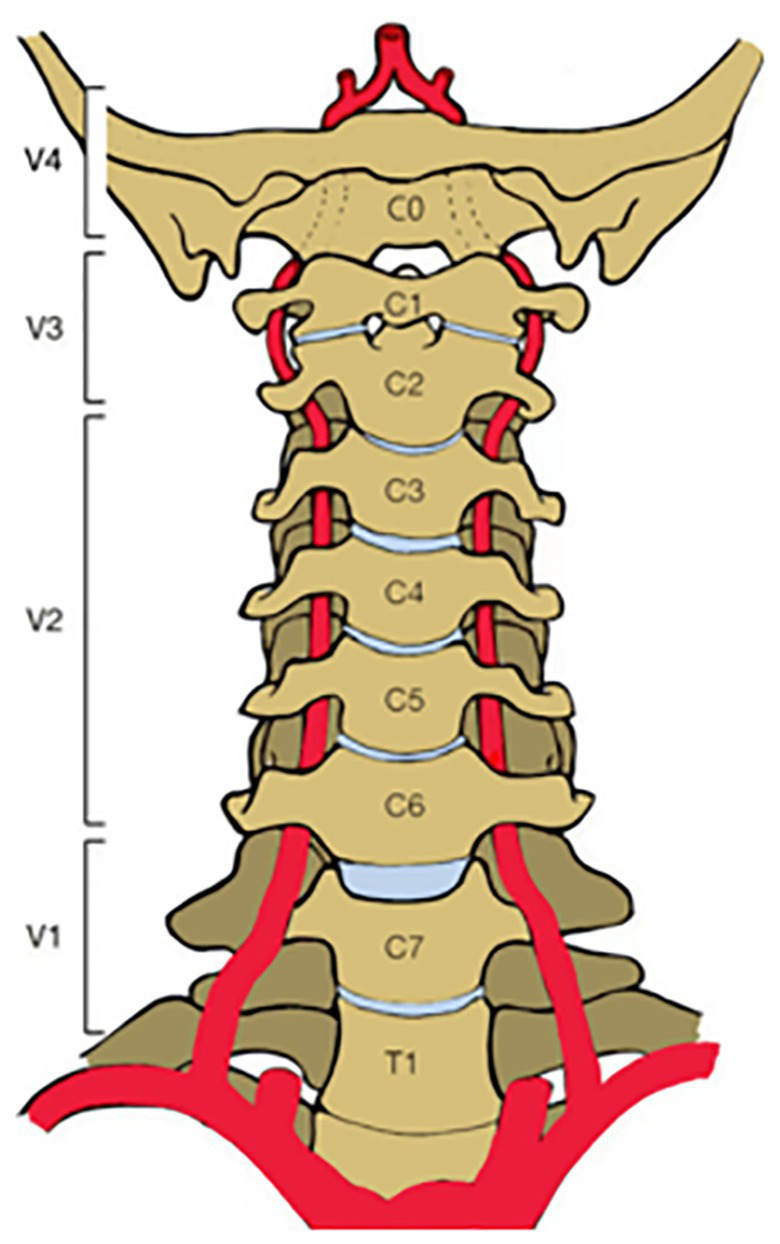
Segmentation of the vertebral artery.

The vertebral arteries branch from the subclavian artery, one on each side of the body, and then extend upward in the transverse foramen of each cervical vertebra, merging to form a single midline basilar artery. The relationship between the vertebral artery and the cervical spine is closed that the anatomy of the VA is often clinically divided into four segments (7): the extraosseous segment between the subclavian artery and C6 (V1 segment); the intervertebral foramen segment through the C6-C3 transverse foramen out of C2 and through the C1 transverse foramen (V2 segment); the extravertebral segment around the atlantoaxial spine out of C1 and ending at the dura (V3 segment) and the intradural segment through the dura to the junction of the basilar artery (V4 segment) (Figure 3). The V3–V4 segment is located in the CVJ, so we focused our study on the V3–V4 segment of the VA.

Export the results of the coordinate range corresponding to the V3–V4 segment via CFD-Post 19.2 and record the following indicators: lumen diameter (D), peak systolic velocity (PSV), mean blood flow velocity (MV), wall pressure (P), wall shear stress (WSS), normalized WSS (NWSS).

### Statistical analysis

SPSS 21.0 software was used for statistical analysis, and data were expressed as mean ± standard deviation ( $\bar{x}$ ± s). Paired samples *t*-test was used for intra-group comparisons, and independent *t*-test was used for inter-group comparisons. Probability values less than 0.05 were uniformly specified as being statistically significant.

## Results

### CJA group vs. control group

The lumen diameter of VA in the CJA group was smaller than that in the control group (*t* = −3.947, *p* < 0.05); the PSV, MV, P, WSS, and NWSS were larger than those in the control group (*t* = 4.361, 3.737, 2.975, 3.360, 2.701, *p* < 0.05) (Table 2).

**Table 2:** Comparison of hemodynamic data between the two groups.

	CJA group	Control group	*t*	*P*
Total cases	60	60		
D (mm)	3.354 ± 0.562	3.744 ± 0.520	−3.947	<0.001*
PSV (m/s)	1.235 ± 0.182	1.104 ± 0.145	4.361	<0.001*
MV (m/s)	0.339 ± 0.063	0.307 ± 0.042	3.737	<0.001*
P (pa)	24576.980 ± 7095.836	20824.281 ± 6718.438	2.975	0.004*
WSS (pa)	34.863 ± 6.816	31.080 ± 5.438	3.360	<0.001*
NWSS	0.272 ± 0.075	0.237 ± 0.067	2.701	0.008*

*All measured hemodynamic parameters were statistically significant in two groups. (*P* < 0.05).

### Case presentation

#### Case 1

**Figure 4:**
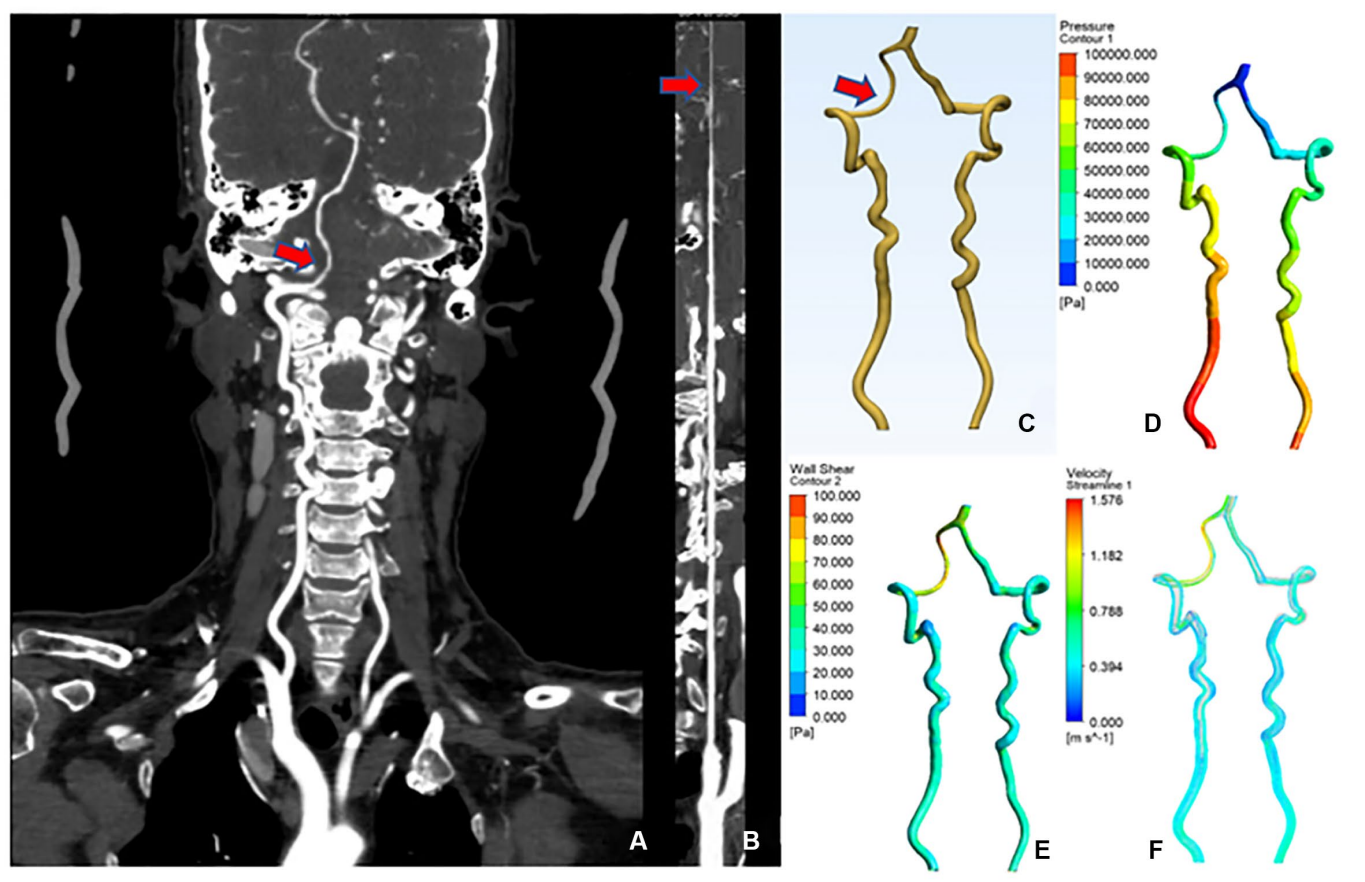
CTA and CFD images of vertebral artery in case 1. **(A)** Vertebral artery CTA coronal position. **(B)** Straightened right vertebral artery. **(C)** CAD model of vertebral artery after smoothing process. **(D)** Changes in wall pressure gradient at the vertebral artery. **(E)** Changes in wall shear gradient at the vertebral artery. **(F)** Vertebral artery flow velocity streamline.

A 60-year-old female was admitted with the chief complaint of “intermittent dizziness with numbness of extremities over 20 years.” A combined head and neck CTA showed: “1. Calcium spots in the wall of the V3 and V4 segments of the right vertebral artery with mild luminal stenosis; 2. Basilar invagination (compression of the right vertebral artery considered).” The patient was diagnosed with “BI,” CFD results showed that the mean lumen diameter of the V3-V4 segment of the VA was lower on the right (2.33 mm) than on the left (3.85 mm); the mean wall pressure was higher on the right (31.20 kPa) than on the left (21.54 kPa); the mean WSS was higher on the right (36.73 Pa) than on the left (41.06 Pa); the mean flow velocity was higher on the right (1.02 m/s) than on the left (0.54 m/s) (Figure 4).

#### Case 2

**Figure 5:**
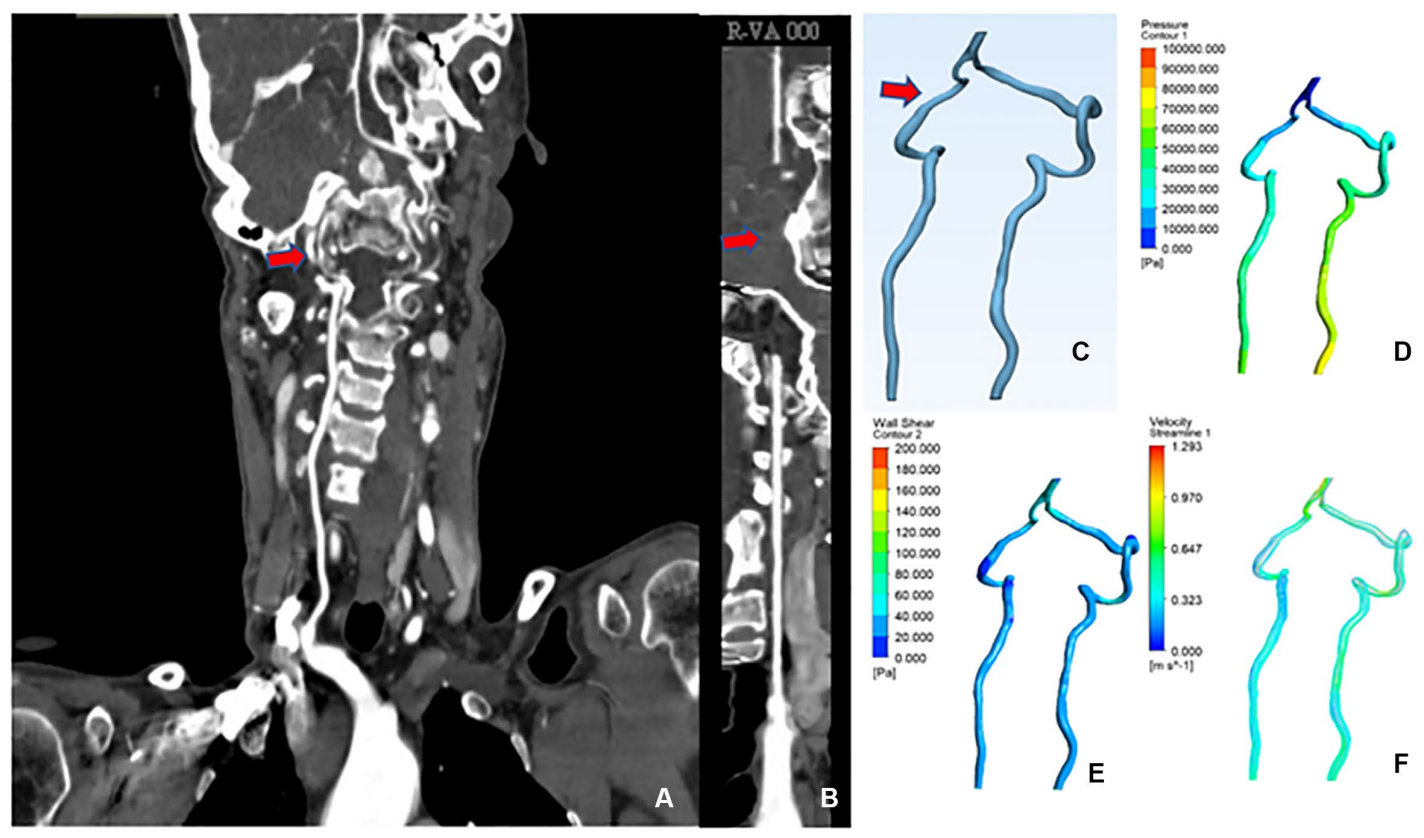
CTA and CFD images of vertebral artery in case 2. **(A–F)** The same as shown in Figure 4.

A 56-year-old female was admitted to the hospital with the chief complaint of “weakness of the limbs over 10 months, persistent vertigo and nausea for 10 days.” The combined head and neck CTA showed that “1. The right vertebral artery V3 segment is tortuous with mild lumen narrowing; 2. Atlantoaxial dislocation, C1 occipitalization, mild basilar invagination, and cervical scoliosis.” The patient was diagnosed with “1.AAD, 2.BI, 3. C1 occipitalization.” CFD results showed that the mean lumen diameter of the V3-V4 segment of the VA was lower on the right (2.04 mm) than on the left (3.44 mm); the mean wall pressure was higher on the right (28.20 kPa) than on the left (19.34 kPa); the mean WSS was higher on the right (28.87 Pa) than on the left (20.10 Pa); the mean flow velocity was higher on the right (0.88 m/s) than on the left (0.32 m/s). The flow velocity curve of the right V3–V4 segment was sparser than that of the left, suggesting increased resistance and decreased blood flow and blood flow in the right V3–V4 segment of the VA (Figure 5).

## Discussion

**Figure 6:**
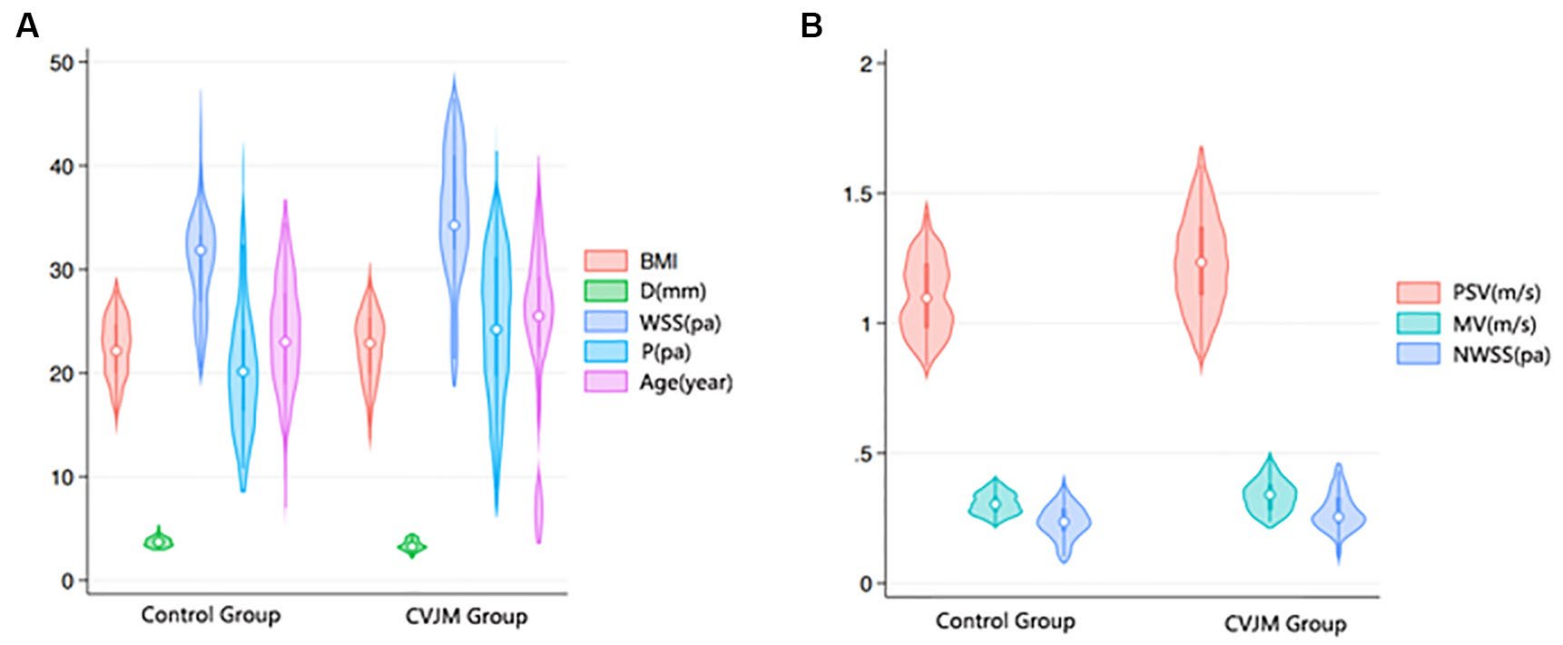
Comparison of age, BMI and hemodynamic-related data between control and CVJM groups. **(A)** Violin Plot of Age, BMI, D, P, and WSS distribution between control and CVJM groups. **(B)** Violin Plot of PSV, MV, and NWSS distribution between control and CVJM groups.

CJA and VA variation occur with each other, and when complex CJA is present, the likelihood of VA variation is higher than in the normal population (8, 9). In this study, we found that in CJA patients, the VA had a large distribution of differences in morphology, alignment, canal diameter, and hemodynamic parameters, whereas in the control group, there were no significant differences in the indexes of the VA (Figure 6), which is consistent with clinical experience and previous studies (10), and provides a theoretical basis for multiple vascular variants such as the VA in patients with CJA.

Previous studies found that both CJA and VA variants are the result of congenital embryonic differentiation abnormalities which is caused by common genetic or environmental factors (11). Anomalies of the bones in CVJ may affect the development and shape of the VA, resulting in VA variation and changing the blood flow of the VA. In addition, the instability of the atlantoaxial joint caused by the CJA makes the VA more vulnerable to external injury or compression during activity. Moreover, the severe bony deformity may lead to impaired cerebrospinal fluid circulation, which leads to increased or unstable intracranial pressure and affecting the perfusion of the VA. Accordingly, when patients have a VA variant, it may lead to reduced or unbalanced intracranial blood flow, affecting intracranial pressure and cerebrospinal fluid circulation, inducing or aggravating CJA such as Chiari malformation. In conclusion, in CJA, there is usually a variation in VA and a higher risk of injury during surgery. A study by Molinari R reported a high incidence of VA injury (up to 8%) was observed in CJA patient (12). As a result, a detailed preoperative understanding of VA alignment, morphology and parameters is particularly important.

The altered hemodynamics of the VA in patients with CJA are closely related to their anatomy abnormality. These alterations may lead to changes in vessel wall stress, increased vascular resistance, and chaotic blood flow, which results in physiological and pathological responses in the vessel wall, such as local inflammation, fibrosis, calcification, hemorrhage, and aneurysm formation. The common sites of vertebral artery stenosis caused by bony deformity mainly occur through the C1 and C2 transverse foramina, and the coordinates of the C1-C2 region were selected to derive the relevant data for calculation during the final flow visualization of CFD results. It was found that V, P, and WSS are mostly elevated before the stenosis site and gradually decrease when the blood flow passes through the stenosis site. In the pre-stenotic region, due to the increased velocity of blood flow, the high flow rate impinges on the wall, generating high wall pressure and wall shear, which is consistent with the previous findings of Qiao et al. (13) on vertebral artery stenosis.

Patients with CJA often compress adjacent anatomical structures leading to the development of vertebral artery stenosis. A narrowed vertebral artery leads to an increased blood flow velocity, which results in turbulent blood flow, blood stagnation and even hypercoagulation, causing the formation of arterial plaque and atherosclerosis. The high flow rate leads to an increase in wall pressure, making the vessel wall surface more vulnerable to injury and thus rupture, which suggests that the surgeons should try to operate as delicately as possible during the surgical operation to avoid damaging the vertebral artery and causing unnecessary consequences. High WSS can stimulate increased NO production in vascular endothelial cells and induce smooth muscle cell (SMC) apoptosis, which leads to arterial plaque formation (14). In severe cases, it leads to the formation of atherosclerosis, which further aggravates the stenosis. Moreover, high WSS often leads to chronic damage to the endothelial cells of the vessel wall (15), thus causing a decrease in the endothelial cells of the arterial vessel wall, increased permeability of the vessel wall, degeneration of the elastic layer, and thinning of the middle layer, which are the main factors in the development and growth of aneurysms (16, 17). This also provides a theoretical basis for the frequent coexistence of CJA with aneurysms in clinical practice. These differences may lead to different physiological responses and disease risks in the hemodynamics, vessel wall physiology and pathology of the malformed vertebral artery from those of the normal human vertebral artery. Therefore, preoperative clarification of vertebral artery abnormalities, especially hemodynamic abnormalities, in patients with CJA is important for the development of treatment plans.

Abnormalities in the vertebral arteries account for a large proportion of the causes of vascular dizziness. Many studies have showed that abnormalities in the carotid arteries are associated with symptoms of dizziness in patients (18, 19). The vertebral artery-basilar artery ring supplies 1/5 of the blood flow to the brain, and stenosis of the VA may lead to hypoxia and ischemia in the brain, resulting in dizziness (20). The stenosis of the VA caused by the CJA makes the blood flow velocity increase. According to the Reynolds coefficient (Re) formula, when the blood flow velocity increases, the blood flow will be turbulent when Re exceeds 4,000, which leads to blood stagnation. At the same time, the blood flow through the unit area is greatly reduced due to the reduction of the canal diameter, which may cause a certain degree of ischemia in the patient’s brain, resulting in the occurrence of clinical symptoms such as dizziness, vertigo, and headache.

The further research direction is to investigate in specific correlation between this hemodynamic difference and the patient’s dizziness symptoms, to assess the patient’s clinical symptoms through the dizziness handicap inventory (DHI) and other scores, and to regress the correlation between various factors on the patient’s dizziness symptoms, so as to provide more basis for the clinical treatment of this type of patients. The three-dimensional reconstruction of the deformed VA using CFD visualization not only makes it easier to visualize the morphology and course of the VA, but also simulates and calculates hemodynamic and other parameters, providing a basis for preoperative evaluation and surgical planning for these patients. The way forward is the need to further evaluate each of the potential pathogenic variables, i.e., the degree of brainstem compression and vertebral artery changes, and to quantify the clinical symptoms utilizing assessment scales to determine if these velocities, diameters, wall pressures, and shear forces have any effect on aneurysm formation and clinical symptoms.

## Limitations

The study was a retrospective analysis study and may have some error due to the low incidence resulting in a small number of cases. (ii) The study excluded patients with congenital malformations of blood vessels, which may lead to some selection bias. (iii) This study assumed a Newtonian fluid blood and rigid vessel walls, which has some deviation from the actual physiological state of blood vessels. (iv) The hypothesis of this study that reduced vertebral artery blood flow is associated with dizziness symptoms in patients with CJA is an ideal situation, and further evidence is needed to prove the correlation.

## Conclusion

In patients with CJA, the hemodynamic differences in diameter, flow velocity, flow rate, wall pressure and shear force of the VA are different from those of the normal population, and may lead to the occurrence of clinical symptoms such as dizziness. When there is a complex CJA, the possibility of VA variation is higher than that of the normal population, especially in patients with dizziness, and preoperative combined head and neck CTA and other related examinations should be performed to clarify the vascular abnormalities. The CFD visualization technology can reconstruct the vertebral artery in three dimensions for analysis of various parameters, which is beneficial for preoperative evaluation and treatment planning.

## Data availability statement

The raw data supporting the conclusions of this article will be made available by the authors, without undue reservation.

## Ethics statement

The studies involving humans were approved by the Ethics Committee of the First Affiliated Hospital of Zhengzhou University. The studies were conducted in accordance with the local legislation and institutional requirements. The participants provided their written informed consent to participate in this study. Written informed consent was obtained from the individual(s) for the publication of any potentially identifiable images or data included in this article.

## Author contributions

YL contributed to the conception and design of the study. ZZ wrote sections of the manuscript. RT, XA, and YX embellished the language. LW, YL, YW, YZ, and SZ critically reviewed and revised the manuscript. RZ analyzed the database. All authors reviewed and approved the final version of the manuscript.
